# The Men Won’t Propose

**Published:** 1866-12

**Authors:** 


### THE MEN WON'T PROPOSE.

Because they are afraid of the enormous expenses of housekeeping. It requires a little fortune, now, to buy a house, and every article of furniture costs about three times as much as it did ten years ago. Young men of spirit, and they are the only ones worth having now, begin to calculate the cost of wedlock. When they see the extravagant lengths to which our daughters go in their dress; when they look at the splendid mansions in which their fathers live, their minds begin to run in this channel; "She is a charming girl, in fact, too good for me, but to place such a trusting creature in a condition iuferior to the one in which she now finds herself, would be dishonorable, and I must forego the happiness of marrying her, even were she willing, until I have obtained the means of placing her in a social position worthy of her;" and while he is bending his energies to bring about this end, years creep on; opinions have changed, views of life have altered; the affections have become chilled and the mind hardened with its attritions with men, preferences have been diverted, and in too many cases an old bachelor and an old maid occupy the places which otherwise might have been the abode of a happy family and a delightful association.

Every body ought to get married who can boast of three things, First, A sound body. Second, A sound mind. Third, A good trade; this as to men; and as to women, they should possess good health, tidiness and industry. With these, any young couple can get as rich as they ought to be, or as rich as is necessary to an enjoyable life, if they will only go to housekeeping a little below their ability.

The young should have the courage to live within their means; to have more pride in the consciousness that they have a little spare money at home, than in living in a style which keeps them all the time cramped in maintaining. Better to live in one room with all the furniture your own than occupy a whole house with scarcely a chair or table paid for.

